# Comment on “Is Acupuncture Effective in Diminishing Frown Lines? Evidence From a Randomized Controlled Trial”

**DOI:** 10.1111/jocd.70663

**Published:** 2026-01-07

**Authors:** Zihan Gao, Xiaoman Liu, Jiachun Xu

**Affiliations:** ^1^ Tianjin University of Traditional Chinese Medicine Tianjin China; ^2^ The Second Affiliated Hospital of Tianjin University of Traditional Chinese Medicine Tianjin China

Dear Editor,

Haghir et al. conduct a pioneering randomized waitlist‐controlled trial investigating the efficacy of combined facial and body acupuncture for frown lines, providing valuable evidence for this understudied cosmetic intervention [1]. The study demonstrates significant improvements in glabellar lines, patient satisfaction, and social functioning, with minimal adverse effects, supporting acupuncture as a safe alternative to invasive or toxin‐based treatments.

However, several methodological considerations warrant attention. First, the control group was significantly younger than the intervention group (*p* = 0.03), a confounding factor given that younger age may inherently favor better skin condition and wrinkle improvement. Second, while outcome assessors and analysts were blinded, patients and the acupuncturist were not, introducing potential performance and expectation biases that could inflate efficacy outcomes. Third, the use of a wait‐list control group does not account for placebo effects, which are particularly significant in nonpharmacological, perceptible interventions like acupuncture. The absence of a sham acupuncture control substantially limits the ability to attribute observed benefits specifically to the physiological effects of needling.

Controversially, the study attributes improvements to mechanisms like collagen synthesis and muscle relaxation but lacks objective measurements (skin elasticity, collagen density) to validate these biological claims, relying instead on subjective scales (GAIS, SSS). Additionally, the combined use of facial, body, and intradermal acupuncture prevents delineation of each component's specific contribution to outcomes.

Overall, this work advances evidence for acupuncture in facial rejuvenation, with strengths in blinded assessment and long‐term follow‐up. Addressing confounding variables, incorporating objective biometric measures, and testing component‐specific effects in future multicenter trials will strengthen conclusions. Nevertheless, the study provides a compelling foundation for considering acupuncture as a safe, patient‐centered option for frown line reduction.

## Author Contributions

All authors wrote the manuscript text. All authors have read and approved the final version of the manuscript.

## Funding

The authors have nothing to report.

## Ethics Statement

The authors have nothing to report.

## Consent

The authors have nothing to report.

## Conflicts of Interest

The authors declare no conflicts of interest.

## Linked Articles

Is acupuncture effective in diminishing frown lines? Evidence from a randomized controlled trial, https://doi.org/10.1111/jocd.70144.

## Data Availability

The data that support the findings of this study are available on request from the corresponding author. The data are not publicly available due to privacy or ethical restrictions.
